# The prevalence and impact of Fusarium head blight pathogens and mycotoxins on malting barley quality in UK

**DOI:** 10.1016/j.ijfoodmicro.2014.03.023

**Published:** 2014-06-02

**Authors:** L.K. Nielsen, D.J. Cook, S.G. Edwards, R.V. Ray

**Affiliations:** a Division of Plant and Crop Sciences, School of Biosciences, The University of Nottingham, Sutton Bonington, UK; b Division of Food Science, School of Biosciences, The University of Nottingham, Sutton Bonington, UK; c Crop and Environment Sciences, Harper Adams University, Newport, UK

**Keywords:** *Fusarium*, *Microdochium*, Malting barley, Mycotoxins, Malting, Brewing

## Abstract

Fusarium head blight (FHB) caused by *Fusarium* and *Microdochium* species can significantly affect the yield of barley grain as well as the quality and safety of malt and beer. The present study provides new knowledge on the impacts of the FHB pathogen complex on the malting and brewing quality parameters of naturally infected barley. Quantitative real-time PCR and liquid chromatography double mass spectrometry were used to quantify the predominant FHB pathogens and *Fusarium* mycotoxins, respectively, in commercially grown UK malting barley samples collected between 2007 and 2011. The predominant *Fusarium* species identified across the years were *F. poae*, *F. tricinctum* and *F. avenaceum*. *Microdochium majus* was the predominant *Microdochium* species in 2007, 2008, 2010 and 2011 whilst *Microdochium nivale* predominated in 2009. Deoxynivalenol and zearalenone quantified in samples collected between 2007 and 2009 were associated with *F. graminearum* and *F. culmorum*, whilst HT-2 and T-2, and nivalenol in samples collected between 2010 and 2011 correlated positively with *F. langsethiae* and *F. poae*, respectively. Analysis of the regional distribution and yearly variation in samples from 2010 to 2011 showed significant differences in the composition of the FHB species complex. In most regions (Scotland, the South and North of England) the harvest in 2010 had higher concentrations of *Fusarium* spp. than in 2011, although no significant difference was observed in the Midlands between the two years. *Microdochium* DNA was significantly higher in 2011 and in the North of England and Scotland compared to the South or Midlands regions. Pathogens of the FHB complex impacted negatively on grain yield and quality parameters. Thousand grain weight of malting barley was affected significantly by *M. nivale* and *M. majus* whilst specific weight correlated negatively with *F. avenaceum* and *F. graminearum*. To determine the impact of sub-acute infections of the identified *Fusarium* and *Microdochium* species on malting and brewing quality of naturally infected samples, selected malting barley cultivars (Optic, Quench and Tipple) were micromalted and subjected to malt and wort analysis of key quality parameters. *F. poae* and *M. nivale* decreased germinative energy and increased water sensitivity of barley. The fungal biomass of *F. poae* and *F. langsethiae* correlated with increased wort free amino nitrogen and with decreased extract of malt. DNA of *M. nivale* correlated with increased malt friability as well as decreased wort filtration volume. The findings of this study indicate that the impact of species such as the newly emerging *F. langsethiae*, as well as *F. poae* and the two non-toxigenic *Microdochium* species should be considered when evaluating the quality of malting barley.

## Highlights

• First report of the impact of naturally occurring FHB species on brewing quality
• Diverse FHB complex comprised of *Fusarium* and *Microdochium* spp. in UK barley
• Strong positive correlations found between mycotoxins and relevant producers
• Toxigenic *Fusarium* and non-toxigenic *Microdochium* reduce yield quality parameters.
• Brewing quality significantly impaired by *M. nivale*, *F. poae* and *F. langsethiae*

## 1 Introduction

Fusarium head blight (FHB) is an important disease of barley (*Hordeum vulgare*) caused by a complex of toxigenic *Fusarium* spp. and non-toxigenic *Microdochium* spp. known to impact significantly upon the yield and several functional parameters of grain related to malting and brewing quality (Sarlin et al., 2007; Schwarz, 2003; Schwarz et al., 2006). Furthermore, several *Fusarium* species produce mycotoxins hazardous to humans and animals if consumed (D'Mello et al., 1999; Desjardins, 2006). *Fusarium graminearum* and *Fusarium culmorum* are potent producers of zearalenone (ZON) and type B trichothecenes, deoxynivalenol (DON) and nivalenol (NIV) (Bottalico and Perrone, 2002). *Fusarium langsethiae* and *Fusarium sporotrichioides* are producers of Type A trichothecenes, HT-2 and T-2 (Thrane et al., 2004). *Fusarium poae* produces NIV and diacetoxyscirpenol (DAS), whereas *F. avenaceum* and *F. tricinctum* are associated with moniliformin, enniatins and beauvericin (Thrane et al., 2004). However, under North European growth conditions *F. avenaceum* mainly produces enniatins (Jestoi et al., 2004; Uhlig et al., 2007; Yli-Mattila et al., 2009). In 2006, the European Commission set legislative limits for the main mycotoxins produced by the *Fusarium* species in cereals and cereal products intended for human consumption (The European Commission 1881/2006). At present, the legislation includes DON and ZON with limits of 1250 ppb and 100 ppb respectively for unprocessed cereals (EC 1881/2006). No legislative limit has been set for NIV as the amount of NIV usually follows closely the levels of DON and thus it is envisaged that the legislation for DON will prevent unacceptable exposure to this toxin (Leslie et al., 2008). New indicative limits for HT-2 and T-2 were published in 2013 as the combined maximum of HT-2 and T-2 toxins of 100 ppb for unprocessed wheat and 200 ppb for unprocessed barley (EC 2013/165/EU).

The immediate effects of severe pre-harvest infection of barley with the species of the FHB complex are reduced seed germination and grain functionality affecting the marketability of the crop and ability to attract malting premium. Further quality problems arise during malting and brewing with severely infected malts being associated with the occurrence of gushing and/or changes in colour and flavour of the finished beer (Oliveira et al., 2012a).

To ensure the quality of barley grain destined for commercial malting and brewing and deemed acceptable for this purpose, the UK malting industry has imposed strict minimum grain specifications which must be met by producers (Assured malt UK, 2008). In addition to quality requirements of acceptable commercial viability of the grain of more than 98% germinative energy (GE), minimum standards include inspection for fungal contamination at intake and due diligence testing for mycotoxins thus preventing heavily infected and quality compromised grain bulks entering the supply chain of malt to beer (HGCA, 2002).

The majority of information on the impact of FHB disease on malting and brewing quality has been provided by artificially inoculated pre- or post-harvest experiments of barley grain and malt using individual *Fusarium* species. These studies have identified *F. graminearum* and *F. culmorum* as the most damaging from the FHB complex, followed by *F. poae* and *F. avenaceum*, impacting on several malting and brewing quality parameters (Schwarz et al., 2001). A field experiment using artificial inoculation of barley heads pre-harvest with *F. graminearum*, *F. culmorum* or *F. poae* showed that inoculation resulted in significant reductions in grain plumpness and germination capacity and a slight increase in protein and nitrogen content in the grain (Sarlin et al., 2005). The same three *Fusarium* species were shown to induce gushing of beer with *F. culmorum* and *F. graminearum* being the most potent inducers (Sarlin et al., 2005). Two malting experiments using barley grain artificially inoculated post-harvest with *F. culmorum* demonstrated an increase in friability, protease and β-glucanase activities, lower amylase activity, greater proportion of free amino and soluble nitrogen and lower β-glucan content, a significant malt loss, as well as a change in the protein content compared to malt from non-infected grain (Oliveira et al., 2012b, 2013). Experiments using artificial inoculation with individual *Fusarium* species provide valuable information on the severity of FHB related issues in the worst case scenario, but they are less representative of commercial crop production situations where barley grain is likely to be infected by more than one causal organism. Indeed, recent surveys of European commercial barley crops have shown that the FHB complex occurring on the crop is much more diverse than previously considered, including, apart from *F. graminearum* (*F. graminearum* sensu stricto), *F. culmorum* and *F. poae*, mixed populations of newly emerging pathogens such as *F. langsethiae*, *F. avenaceum*, *F. tricinctum* and *Microdochium nivale* and *Microdochium majus* (Nielsen et al., 2011, 2013). Thus, the cumulative impact of the FHB species complex and their related mycotoxins in naturally infected barley upon the malting and brewing quality parameters within limits of acceptable malting capability has not been previously investigated. Furthermore, there is no published information on the effects of *F. langsethiae*, a potent HT-2 and T-2 producer in barley, or non-toxigenic species such as *Microdochium* occurring in temperate geographical locations. Here we report on the distribution, co-occurrence and impacts of diverse FHB fungal communities in commercially grown barley crops. Quantitative real-time PCR (QPCR) and LC/MS/MS were applied to quantify pathogen DNA and mycotoxin concentrations, respectively, and a sub-set of the survey samples was subjected to micromalting and laboratory mashing analysis in order to determine potential quality impacts related to naturally occurring mixed fungal loads and mycotoxins.

The present study is based on two annual surveys of commercially grown UK spring malting barley varieties collected in 2010 and 2011, as well as UK spring barley samples collected as part of a previous mycotoxin survey between 2007 and 2009. The main objectives of this study were i) to identify and quantify the main species of the FHB complex and their related mycotoxins in naturally infected field samples of UK malting barley, ii) to determine the regional distribution and co-occurrence of the predominant species associated with FHB disease in UK, iii) to assess the influence of known agronomic factors on fungal populations and iv) to quantify the cumulative impact of fungal and/or mycotoxin contamination on the malting and brewing quality parameters of barley grain as close as possible to commercial malting standards for grain viability.

## 2 Materials and methods

### 2.1 Malting barley grain samples

A total of 228 samples of malting barley of commonly grown varieties were collected at harvest between 2007 and 2011 from UK fields. Malting barley samples (n = 63) from harvests 2007–2009, which contained a known range of mycotoxins, were obtained from a previous project studying the occurrence of *Fusarium* mycotoxins in malting barley (Edwards, 2012). During 2010 and 2011 malting barley samples (n = 165) and limited agronomy data including region and barley cultivar, were provided from commercially grown barley fields in UK. A summary of the distribution of the samples included in this study based on sampling year, numbers used in analysis, sampling region and variety is shown in Tables A.1 and A.2.

### 2.2 DNA extraction

Barley grain samples (2 kg) collected at harvest were mixed manually and divided into sub-samples for microbiological, molecular, mycotoxin and brewing analysis. Two hundred grams of each sample was milled (ZM100, Retsch UK Ltd., Leeds, with a 1 mm screen) and stored at − 20 °C until DNA extraction. Flour samples (4 g) were weighed individually into 50 ml tubes and 30 ml CTAB buffer (87.7 g NaCl, 23 g sorbitol, 10 g N-lauryl sarcosine, 8 g hexadecyl trimethylammonium bromide, 7.5 g ethylenediamine tetraacetic acid and 10 g polyvinylpolypyrolidone, made up to 1 l with distilled water) was added. The contents were mixed and incubated at 65 °C for 2 h. Ten millilitres of 5 M potassium acetate was added to the tubes, which was then mixed and stored at − 20 °C overnight. Samples were thawed and centrifuged at 3000 ×*g* for 15 min. A 1.2 ml volume of supernatant was transferred to a sterile 2.0 ml Eppendorf tube, then chloroform (0.6 ml) was added and the contents mixed for 1 min and centrifuged at 11,000 ×*g* for 15 min. A portion of the aqueous phase (1 ml) was removed and transferred to a new sterile 2.0 ml Eppendorf tube containing isopropanol (0.8 ml), mixed for 1 min and placed for 1 h at − 20 °C. The samples were centrifuged at 12,000 ×*g* for 15 min and the resulting DNA pellets were washed twice with 1 ml of 44% isopropanol. Pellets were air dried and resuspended in 0.2 ml TE buffer and incubated at 65 °C for 2 h. The samples were vortexed and centrifuged at 12,000 ×*g* for 5 min. DNA was measured and quantified based on absorbances at 260 nm, 280 nm, 328 nm and 360 nm using a Cary® 50 spectrophotometer (Varian, CA, USA) and diluted to a working stock of 20 ng/μl and stored at − 20 °C.

### 2.3 Quantitative real-time PCR

Morphological identification of FHB related species on selected barley samples from 2010 was performed according to the procedures described in the *Fusarium* Laboratory Manual (Leslie and Summerell, 2006) to determine the most commonly occurring species for later quantification by QPCR.

All malting barley DNA samples were analysed using QPCR to quantify the species of the FHB complex found to be the most frequently occurring in these samples by morphological identification. Amplification and quantification of the relevant species in the malting barley flour samples were performed using a real-time PCR thermal cycler CFX96 (Bio-Rad, UK). Pure DNA isolates, which had been identified and verified by both morphological and molecular methods, of *F. graminearum* (isolate 212, University of Nottingham), *F. culmorum* (isolate 236, University of Nottingham), *F. avenaceum* (isolate 40, University of Nottingham), *F. tricinctum* (isolate 53, University of Nottingham), *F. poae* (isolate 246, University of Nottingham), *F. langsethiae* (isolate 227, University of Nottingham), *M. nivale* (isolate 226, University of Nottingham) and *M. majus* (isolate 224, University of Nottingham) were used to make DNA standard curves (10^0^–10^− 6^ ng/μl). The amplification mix for each species consisted of 250 nM of each primer (forward and reverse) and 2 × iQ SYBR Green Supermix (Bio-Rad, UK) reagent which was used according to manufacturer's instructions. The volume of DNA sample in the reactions was 2.5 μl in a total reaction volume of 12.5 μl. In the negative control 2.5 μl of PCR-grade water replaced the DNA template. The detection limit for all eight assays is 10^− 4^ pg/ng total fungal DNA and all assays had an efficiency of E = 95–103%. Species specific primers used for quantification of the species of interest, assay efficiency and references are presented in Table A.3.

### 2.4 Mycotoxin analysis

Mycotoxin analysis on all samples was performed by Campden BRI (Chipping Campden, UK) using UKAS accredited procedures. The trichothecenes (DON, NIV,HT-2 and T-2) and zearalenone were extracted from flour samples (25 g) into an acetonitrile/water mixture with further clean-up of the trichothecenes by solid phase extraction by passing the filtrate through Bond Elut Mycotoxin SPE columns (Agilent Technologies, Germany) (Klotzel et al., 2006). The LC/MS/MS analysis was performed on an Agilent 1200 Infinity LC system (Agilent Technologies, Germany) with a binary pump, coupled with Agilent 6490 MS/MS ESI (Agilent Technologies, Germany) and equipped with an analytical column Agilent Poroshell 120 EC-C18 (2.1 × 100 mm, 2.7 μm, ID Agilent Technologies, Germany). The flow rate was set at 0.2 ml/min and the injection volume was 10 μl. Mobile phase A was water with 0.2% acetic acid and 5 mM ammonium acetate, mobile phase B was methanol with 0.2% acetic acid and 5 mM ammonium acetate. A linear binary gradient was applied from 20 to 70% phase B within 30 min. The content of phase B was then lowered to 20% within a minute followed by equilibration of the column for 10 min. Quantitative determination of all compounds was performed by operating the mass spectrometer in ESI positive and negative ionisation modes (Schuhmacher et al., 2005).

The quantification of the samples was carried out using matrix-matched standards prepared in-house. Spiked samples were included in each batch to determine extraction recovery. The method had an acceptable recovery range for each trichothecene of 60–120%. The results were corrected for recovery.

The expanded measurement of uncertainty was calculated using a standard coverage factor of 2, equivalent to a confidence of approximately 95% in that the actual level of the mycotoxin being measured lies within the quoted range. The expanded measurement of uncertainty was calculated to be 16% for DON and 13% for ZON. The LOQ for the trichothecenes was 10 ppb and for ZON was 2 ppb. Samples below the LOQ were entered as (LOQ) / 2 in the calculation of mean values.

### 2.5 Grain quality assessment

All samples were assessed for the determination of grain quality parameter thousand grain weight (g, TGW) and specific/hectolitre weight (kg/hl, SPW).

### 2.6 Germinative energy (GE)

GE (4 ml) and GE (8 ml) counts were conducted according to European Brewery Convention (EBC) standard methods (Analytica-EBC, Method 3.6.2). Water sensitivity was calculated from the difference between the 4 ml and 8 ml counts, expressed as a percentage.

### 2.7 Micromalting of barley samples

Fifty four samples of the most commonly UK grown malting barley varieties from the 2010 and 2011 harvests (27 drawn from each) were selected for malting and subsequent malting and brewing quality analyses. The samples were selected on the basis of their germinative energy (GE). Barleys with GE (4 ml) counts down to 80% were used. The samples were further selected on the basis of barley cultivar, known variations in fungal DNA (*Fusarium* and *Microdochium* spp.) and mycotoxin concentration. These samples included 26 of cultivar Tipple, 17 of cv Quench and 11 of cv Optic.

Samples (350 g) were malted in a Custom Lab Micromaltings K steep-germinator and kiln (Custom Laboratory Products, Keith, UK). A manual steeping programme using individual polypropylene tubs was developed so that the steep water was not shared between different samples with different grain microflora. The tubs were floated on the automatically filled steep water in the chamber so that the micromaltings controlled temperature through steeping. Germination and kilning stages were automated. Key process parameters were as follows: steeping: 800 ml of temperate steep water was added to 350 g barley during each steep. Temperature was 16 °C throughout and manual water changes were used to create a ‘3-wet’ steep cycle as follows: 8 h wet — 16 h dry — 8 h wet — 16 h dry — 2 h wet. Germination: samples were transferred to individual malting ‘cages’ and germinated at 16 °C for 4 days, with automatic turning of the sample cages set at 1 min every 10 min. Kilning: the air on temperature cycle during drying was as follows: 55 °C for 8 h, 65 °C for 10 h, 75 °C for 2 h, and 80 °C for 2 h.

### 2.8 Malt analyses

Malt moisture content was measured according to Analytica-EBC, Method 4.2.

Malt friability was measured according to Analytica-EBC Method 4.15 using a Pfeuffer Friabilimeter (Pfeuffer GmbH, Kitzingen, Germany) loaded with 50 g of malt and operated for the standard 8 min. The equipment was calibrated using EBC standard malt samples. Malt α-amylase (dextrinising units, DU) was measured using the Ceralpha Megazyme kit and Malt β-amylase was measured using the Betamyl-5 kit (Megazyme, Bray, Ireland).

### 2.9 Laboratory mashing and associated analyses

Finely ground (0.2 mm screen) malt (50 g) was mashed using a 1-Cube R8 laboratory mash bath (1-Cube, Havlickuv Brod, Czech Republic) according to Analytica-EBC 4.5.1 (congress mash). Laboratory wort filtration volume was measured according to the method of Evans et al. (2011). After returning the first 100 ml of wort collected during laboratory wort filtration, the volume of wort filtered in the next 25 min was measured as an index of mash filterability. The resulting bright worts were analysed for: hot wort extract using an Anton Paar DMA 4500 density metre according to Analytica-EBC Method 4.5.1, free amino nitrogen (FAN) by the spectrophotometric ninhydrin method (Analytica-EBC Method 4.10), wort viscosity according to Analytica-EBC Method 8.4 and EBC wort colour according to Analytica-EBC Method 4.7.1.

### 2.10 Data analysis

All data, apart from malting and brewing data, were analysed using Genstat® Version 14.1 for Windows (VSN International Ltd., UK). Relationships between pathogen DNA and mycotoxins were analysed using single linear regression analysis. Multiple linear regression with groups was used to identify relationships between the DNA of *Fusarium* spp. and *Microdochium* spp. and quality parameters of barley grain such as TGW and SW. Where necessary DNA or mycotoxin data were log_10_ transformed to normalise residual distributions.

Unbalanced analysis of variance, using linear regression was carried out on fungal and mycotoxin data from 2010 to 2011 to determine the significance (P < 0.05) of sampling region and malting barley variety. It was not possible to include data from 2007 to 2009 in this analysis as samples from these years were not randomly selected but on the basis of their known mycotoxin contents. Therefore descriptive statistics were used for the DNA, mycotoxin and malting/brewing data on all selected samples. The DNA of *Fusarium* spp. and *Microdochium* spp. and malting/brewing parameters of samples is presented as mean with 95% confidence intervals and the mycotoxin data is presented as mean with 95th percentile and maximum values. The co-existence of the species of the FHB complex was explored using Principal Component Analysis (PCA) on the correlation matrix of eight variables. These variables were fungal biomass (log_10_ pg/ng of total DNA) of *F. graminearum*, *F. culmorum*, *F. poae*, *F. tricinctum*, *F. avenaceum*, *F. langsethiae*, *M. majus* and *M. nivale*.

Malting and brewing quality data were entered retrospectively into a d-optimal factorial design space using experimental design software (Design Expert, v 7.0, Stat-Ease, Mn, USA). The malting and brewing quality parameters for the 54 barley samples were entered as responses and modelled against 15 factors, which were: the DNA contents of the individual species analysed in the samples for two *Microdochium* and six *Fusarium* species (QPCR data), the barley cultivar, harvest year and the concentrations of five mycotoxins analysed in the samples (NIV, DON, HT-2, T-2, ZON). Modelling progressed via progressive factor reduction, successively removing factors which were of least significance in derived models, until a significant model resulted with factors each of which was significant (P < 0.05) and the model R^2^ was maximised. Interactions between factors were included in models where significant.

## 3 Results

### 3.1 *Fusarium* spp. and *Microdochium* spp. in UK malting barley 2007 to 2011

Species specific QPCR assays were used to quantify *Fusarium* spp. and *Microdochium* spp. in UK malting barley samples collected between 2007 and 2011, data presented in Table 1 as mean value with 95% confidence intervals and incidence (%) for each species. When considering the amount of DNA of the eight quantified species of the FHB complex, the non-toxigenic *M. majus* was the predominant species in samples collected in 2007, 2008, 2010 and 2011 whereas *M. nivale* was the predominant species in 2009. *F. poae* was the main *Fusarium* species in 2007, 2008 and 2009, whereas *F. tricinctum* predominated in 2010 and *F. avenaceum* predominated in 2011. The incidence of the species was calculated according to the presence of DNA in all samples throughout the study and the most frequently occurring species in the majority of the analysed samples were *F. avenaceum* (100%), followed by *M. nivale* (96%), *M. majus* (90%) and *F. poae* (90%). Less frequently occurring species were *F. tricinctum* (81%), *F. langsethiae* (65%), *F. graminearum* (46%) and *F. culmorum* (36%).

### 3.2 Principal component analysis (PCA)

**Fig. 1:**
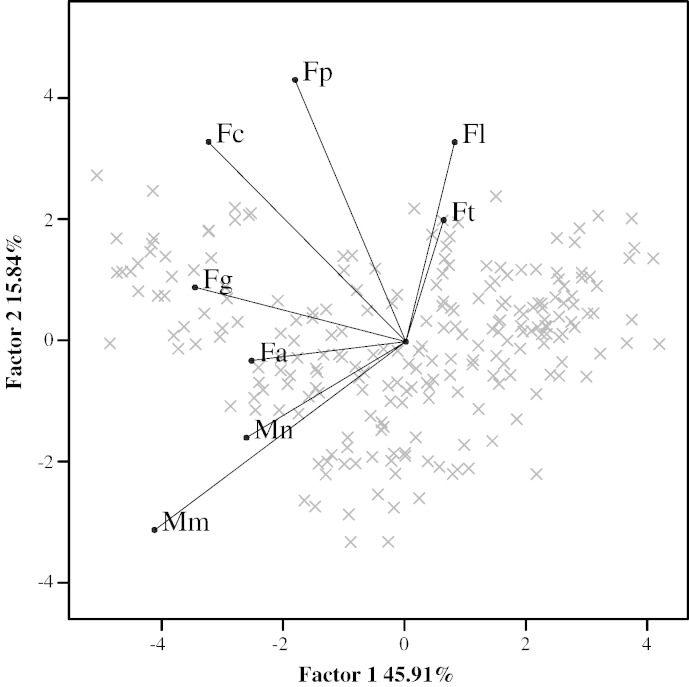
Biplot of the principal component analysis of the quantified amount of fungal DNA in 2010 and 2011 of *F. langsethiae* (Fl), *F. poae* (Fp), *F. culmorum* (Fc), *F. graminearum* (Fg), *F. avenaceum* (Fa), *M. nivale* (Mn) and *M. majus* (Mm).

Quantified DNA of the *Fusarium* spp. and *Microdochium* spp. in samples collected in 2010 and 2011 (n = 151) are plotted as a biplot in Fig. 1. This shows both the distribution of the samples in the two most descriptive dimensions of data and the variables (species) projected onto these two axes. On the x-axis, Factor 1 describes 45.91% of the variability and, on the y-axis, Factor 2 describes an additional 15.84% of the original variability. From the principal component analysis, the co-existence of the different species of the FHB complex is visualised in four clusters. The first cluster consisted of *M. majus* and *M. nivale*, the second of *F. avenaceum* and *F. graminearum*, the third consisted of *F. culmorum* and *F. poae* and a fourth cluster consisted of *F. langsethiae* and *F. tricinctum*. From the PCA analysis, it is evident that there is a strong association between the occurrences of *M. nivale* and *M. majus* and a distinctive negative association between the *Microdochium* group and the cluster of *F. langsethiae* and *F. tricinctum*.

### 3.3 *Fusarium* mycotoxins in UK malting barley 2007 to 2011

The results from the mycotoxin quantification by LC/MS/MS of a total of 143 samples from 2007 to 2009 and selected samples of 2010 (35) and 2011 (45) are presented in Table 2 as mean value, 95th percentile and maximum value. DON, ZON and NIV predominated in the samples collected between 2007 and 2009, however only one sample exceeded the legislative limits of DON of 1250 ppb. No samples exceeded the proposed indicative limit for HT-2 and T-2 of 200 ppb in unprocessed barley. The highest concentration of NIV (1089 ppb) was found in 2011. High ZON concentrations were seen in samples from 2007 to 2008 and 2009. However, it should be noted that these samples were selected retrospectively based on their known mycotoxin content (including DON) and were not randomly collected as in the years of 2010 and 2011. In 2007 four (33%) samples exceeded the legislative limits of 100 ppb. In 2008 and in 2009 eleven (37%) and four (19%) samples respectively exceeded the limits for ZON. All samples in 2010 and 2011 had ZON concentrations below legislative limits.

### 3.4 Relationships between *Fusarium* DNA and mycotoxin concentrations

**Fig. 2:**
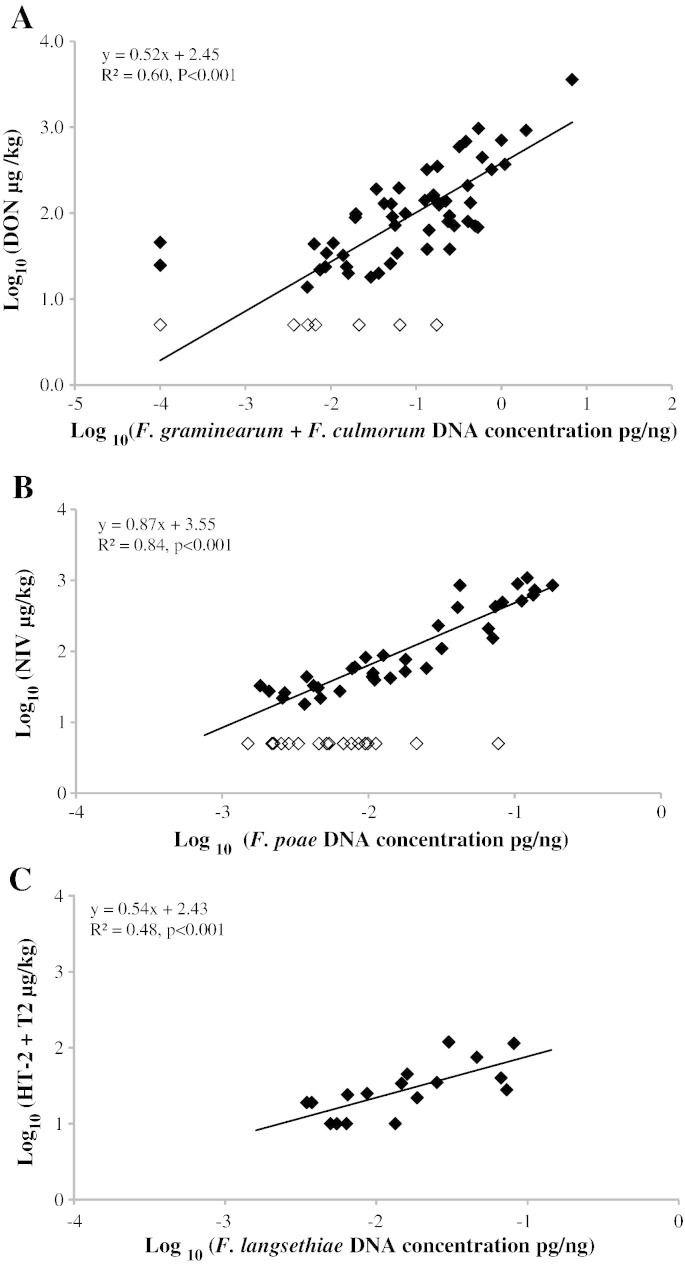
Regression of *Fusarium* species DNA and their corresponding mycotoxins. (A) Regression of log_10_*F. culmorum* and *F. graminearum* on log_10_ deoxynivalenol (DON) in UK barley grain flour samples from 2007 to 2009 (d.f. = 58). Samples below the mycotoxin LOQ are shown without fill and were not included in the regression analysis. (B) Regression of log_10_*F. poae* DNA on log_10_ nivalenol (NIV) in UK barley grain flour samples from 2010 to 2011 (d.f. = 72). Samples below the LOQ are shown without fill and were not included in the regression analysis. (C) Regression analysis of log_10_*F. langsethiae* DNA on log_10_ HT-2 and T-2 quantified in barley samples from 2010 (d.f. = 15). Only samples above LOQ for HT-2 and T-2 were included.

Regressions of mycotoxin concentrations on the quantified *Fusarium* DNA in the analysed barley samples were carried out to identify the main producers associated with grain contamination. All samples above the limit of quantification of individual mycotoxins by the LC/MS/MS assay were used in the regression analysis and samples below the limit of mycotoxin quantification were excluded from the analysis. All regressions of mycotoxins on individual or mixtures of species fitted common lines for the data from individual years suggesting that the relationship between *Fusarium* mycotoxins and their producers is consistent across seasons. A significant positive relationship was observed between the total amounts of *F. graminearum* and *F. culmorum* DNA and the amount of DON in the analysed barley grain samples from 2007 to 2009 accounting for 60% of the variance (P < 0.001, R^2^ = 0.60, d.f. = 58) (Fig. 2A). Regressing DNA of individual species accounted for less of the variance, 41% for *F. graminearum* and 28% of the variance for *F. culmorum* (data not presented). A similar significant relationship (P < 0.001) was between the total amount of *F. graminearum* and *F. culmorum* DNA and ZON accounting for 40% of the variance (data not presented). Analysing *F. graminearum* and *F. culmorum* individually showed that both species were equally similarly associated with ZON but accounted individually for only 30% of the variance (data not presented). *F. poae* DNA showed a significant positive relationship with NIV (P < 0.001, R^2^ = 0.84, d.f. = 72) with 73 of the samples from the 2010 to 2011 harvests fitting a common linear regression (Fig. 2B). A significant positive relationship was also found in 2010 between *F. langsethiae* with total amounts of HT-2 and T-2 (P < 0.001, R^2^ = 0.48, d.f. = 15) (Fig. 2C). All positive samples (16) with HT-2 and T-2 above LOQ were included in the regression analysis and all samples contained *F. langsethiae*.

### 3.5 Effect of cultivar, region and season on the occurrence of FHB pathogens and Fusarium mycotoxins in barley

**Fig. 3:**
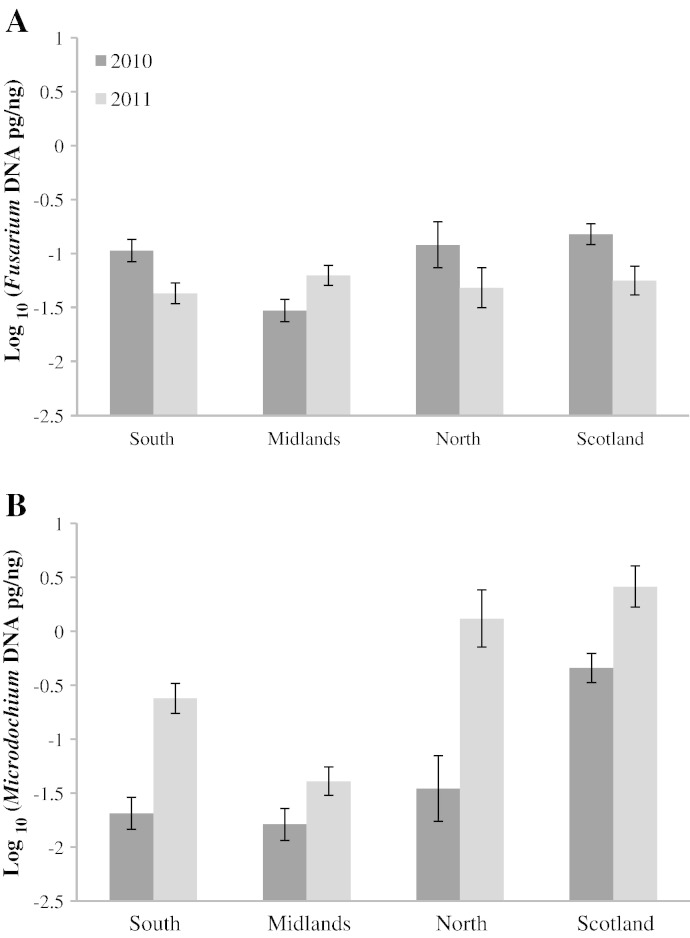
Regional variation of (A) total *Fusarium* spp. DNA and (B) total *Microdochium* spp. DNA determined by quantitative real-time PCR in UK malting barley from 2010 to 2011, described as log_10_ of total DNA (pg/ng) according to a region of collection in UK; higher amounts of *Fusarium* DNA in the South, North and in Scotland in 2010 compared to 2011 (P < 0.001). A). Higher *Microdochium* DNA concentrations in 2011 than in 2010 (P < 0.001, B) and in the North of England and in Scotland compared to the rest of the regions for both seasons (P < 0.001, B). se error bars indicate ± one standard deviation.

The regional and seasonal differences in the amounts of total *Fusarium* DNA and total *Microdochium* spp. DNA found in UK (South, Midlands, North and Scottish) malting barley samples from 2010 to 2011 are shown in Fig. 3A and B respectively. Significantly higher concentrations of *Fusarium* species were found in the South of England and in Scotland in 2010, however there were no significant differences between years for the Midlands. In the North of England, *Fusarium* DNA was found in greater amounts in 2010 than in 2011 (Fig. 3A). The analysis of the regional distribution of *Microdochium* species showed that *Microdochium* DNA increased significantly in 2011 in all regions but was found consistently in highest concentrations in the North of England and in Scotland (Fig. 3B).

**Fig. A.1:**
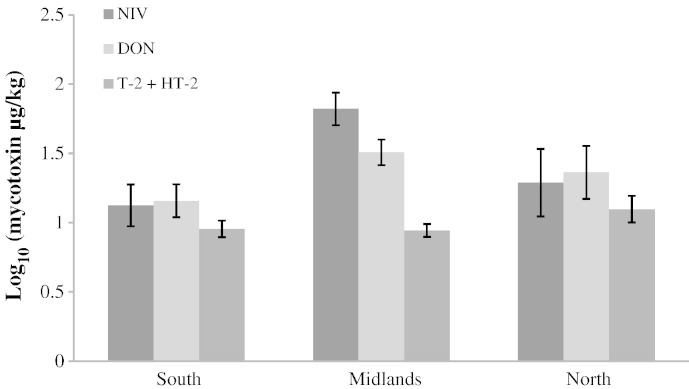
Regional variation of nivalenol (NIV), deoxynivalenol (DON) and T-2 + HT-2 in UK malting barley samples collected in 2010 and 2011. Significant regional differences in NIV (P < 0.001) and DON (P = 0.024). No significant regional difference in T-2 + HT-2 (P = 0.671).

**Fig. 4:**
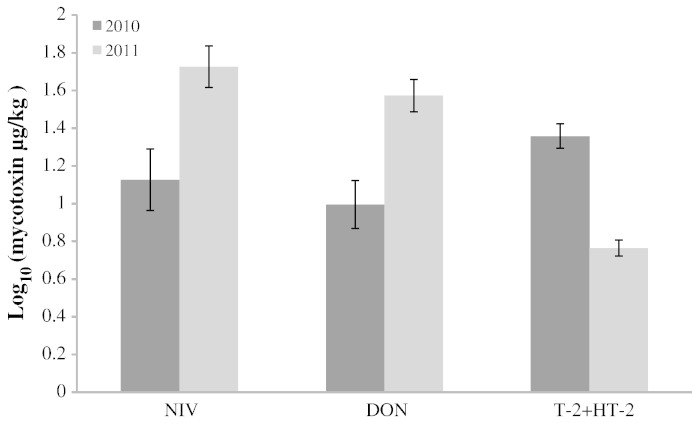
Seasonal variation of nivalenol (NIV), deoxynivalenol (DON) and T-2 + HT-2 in UK malting barley samples collected in 2010 and 2011. Significant seasonal variation for all mycotoxins; NIV (P = 0.012), DON (P < 0.001), T-2 + HT-2 (P < 0.001).

The quantified amounts of the mycotoxins NIV, DON and T-2 + HT-2 showed significant seasonal variation (Fig. 4). In contrast to HT-2 and T-2, which were significantly higher in 2010, NIV and DON increased in 2011. Regional variations were also seen in the distribution of NIV and DON with significantly higher levels of both toxins in the Midlands compared to the South (Fig. A.1).

**Fig. 5:**
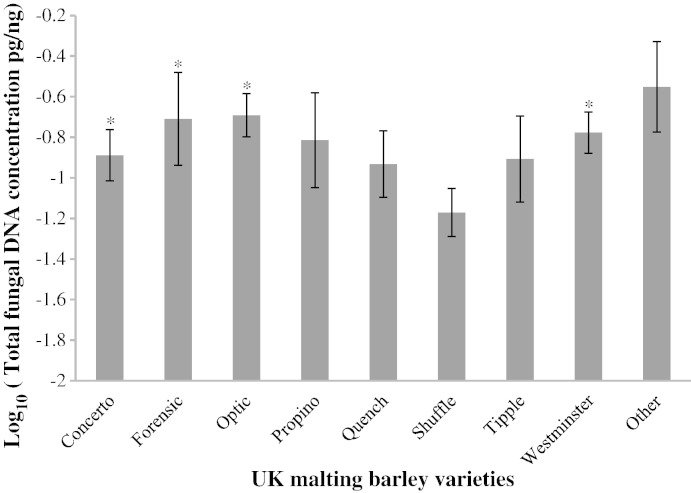
Varietal difference in the amount of fungal DNA in varieties of UK malting barley collected in 2010–2011 described log_10_ of total fungal DNA. Error bars indicate ± one standard deviation. * Indicate varieties significantly different from Shuffle.

Cultivar and regional data of each collected sample were analysed to identify the impact of these parameters on the concentration of *Fusarium* and *Microdochium* spp. Fig. 5 shows the differences in total fungal DNA of *Fusarium* spp. and *Microdochium* spp. quantified in commonly grown commercial cultivars of malting barley collected in 2010 and 2011. There were no significant seasonal effects or interactions between season and cultivar. Cv Shuffle was the only variety which contained significantly lower amounts of total fungal DNA compared to cv Concerto, cv Forensic, cv Optic and cv Westminster (P = 0.042, n = 150).

### 3.6 Effect of FHB related species on grain quality parameters

Multiple linear regressions with groups were used to analyse the relationships between grain quality parameters such as thousand grain weight (TGW; g) and specific weight (SW; kg/hl) and the DNA of individual *Fusarium* and *Microdochium* species in the collected barley samples from different years. Only grain samples with sufficient grain numbers available for analysis were included in the regression analysis. Regression of TGW (d.f. = 177) on DNA of *M. majus*, *M. nivale* and *F. avenaceum* were significant and fitted separate, non-parallel lines for each season (different slopes and intercepts) accounting for 40% of the variance (Table 3). Regression of SW (d.f. = 64) on the DNA of *F. avenaceum* and *F. graminearum* fitted separate but parallel lines (different intercepts) for each season (Table 3). The lines were with negative slopes for all seasons, accounting for 48% of the variance.

### 3.7 Malting and brewing quality parameters

A summary of analytical data for the micromalted samples (n = 54) for each barley cultivar, Optic, Tipple and Quench, and season 2010 and season 2011 is presented as mean and 95% confidence interval in Table 4. Cv Optic and cv Quench produced malts with a greater friability than was observed for cv Tipple using the same micromalting programme. Within each cultivar, the friabilities of malts prepared from the 2010 harvest were somewhat higher than in 2011. In accordance with this, malt α-amylase dextrinising units (DU) were higher on average for malts from the 2010 harvest. The laboratory wort filtration volume (ml) followed similar trends in both 2010 and 2011 with the highest volumes obtained when filtering cv Optic worts, followed by cv Tipple and cv Quench. Laboratory wort viscosity (mPa·s) was higher in 2011 than in 2010 for cultivar Tipple only. This is in accordance with the observed lower friability of Tipple malts prepared in 2011. Laboratory wort colour (EBC) followed the same trends in both 2010 and 2011 with cv Quench producing slightly darker wort colours than cv Optic and cv Tipple.

### 3.8 Modelling of malting and brewing quality data

**Fig. 6:**
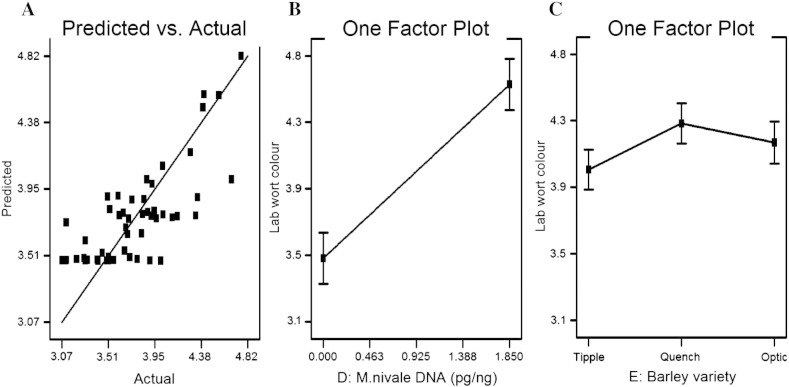
Design Expert plots derived from the model for wort colour. A: predicted wort colour versus actual wort colour; B & C: one factor plots indicating the impacts of (B) *M. nivale* DNA and (C) barley variety on the predicted wort colour.

Wort viscosity and malt β-amylase data for the selected 54 samples failed to reveal any significant trends when modelled against the 15 specified factors (season, barley cultivar, DNA of individual species and mycotoxin data). However, for the remaining parameters, significant models were derived which could satisfactorily predict variations in each parameter across the design space (Table 5). The models with the best predictive power (highest model R^2^; Table 5) were those for water sensitivity of the barley and for laboratory wort colour. The DNA of the individual species identified as significant model terms (Table 5) were those for *F. poae* (GE (4 ml), water sensitivity, laboratory wort extract and wort FAN), *F. langsethiae* (laboratory wort extract and wort FAN) and *M. nivale* (GE (4 ml), water sensitivity, malt friability, laboratory wort filtration volume and laboratory wort colour). The directionality of the factor effects is indicated in Table 5 with a (+) or (–). For example, the increased presence of pathogen DNA for *F. poae* and *M. nivale* correlated with a reduction (–) in GE (4 ml) counts and an increase (+) in water sensitivity. Model data for laboratory wort colour is shown in Fig. 6. Laboratory wort colour was the best fitting predictive model with a reasonable correlation between predicted and actual wort colour values. The factor plots indicate that the action of increasing *M. nivale* concentration was to increase laboratory wort colour by approximately 1 EBC colour unit across the range of concentrations encountered (Fig. 6B) whilst there were significant differences between the barley cultivars Quench and Tipple for wort colour (Fig. 6C).

The analytical concentrations of mycotoxins (NIV, DON, ZON, HT-2 and T-2) and the species DNA data of certain species (*F. avenaceum*, *F. tricinctum*, *F. graminearum*, *F. culmorum*, *M. majus*) were not found to be significant factors in any of the models developed. Of the mycotoxins, NIV was the closest to approaching significance (P < 0.05) in some models. However it co-varied closely with *F. poae* (the main NIV producer) and the models were, in each case, stronger when modelled against *F. poae* DNA rather than the NIV concentrations.

## 4 Discussion

### 4.1 The occurrence of FHB pathogens in UK malting barley

This is the first study using commercially grown, naturally infected malting barley to investigate the cumulative impact of diverse populations of FHB pathogens and their mycotoxins on malting and brewing quality parameters. The findings show that the naturally occurring composition of species of the FHB complex on malting barley in UK is diverse and dominated by non-toxigenic *Microdochium* species and the toxigenic *Fusarium* species *F. poae* and *F. avenaceum*. The presence and amount of species DNA showed yearly variation. *M. majus* was the dominant species in 2007, 2008, 2010 and 2011 and *M. nivale* in 2009. Relatively lower amounts of *F. langsethiae*, *F. graminearum* and *F. culmorum* were found in all five years. Similarly, a survey of the composition of the FHB complex in Danish barley, grown under similar environmental conditions as in the UK, between 2005 and 2007 found that *M. majus* was the predominant species followed by *F. langsethiae*, *F. avenaceum* and *F. poae* (Nielsen et al., 2011, 2013). The predominance of *F. avenaceum* in barley has also previously been shown in Finnish barley (Yli-Mattila et al., 2004). *F. graminearum* is typically considered to be the most prevalent and aggressive FHB pathogen on both wheat and barley in much of the world, particularly in the temperate and warmer regions of the USA, China and the southern hemisphere, whereas *F. culmorum* was associated with FHB in cooler regions such as UK, Northern Europe and Canada (Osborne and Stein, 2007). However, our findings together with the recent work in Europe (Nielsen et al., 2011, 2013; Yli-Mattila et al., 2008) strongly suggest that *F. graminearum* and *F. culmorum* are not the most important pathogens, and particularly *F. culmorum* is occurring less, as part of the FHB complex in barley. This is of particular importance in European locations where research focus should be directed towards understanding the impact of other species previously considered less aggressive but still economically important due to their association with FHB disease and mycotoxin accumulation.

PCA identified clear groupings of co-occurring pathogen species (Fig. 1). Similar to previous studies (Nielsen et al., 2013), *F. culmorum* was found to associate more closely with *F. poae*, whereas a negative association was found between the cluster of *F. langsethiae* and *F. tricinctum* and the cluster of *Microdochium* species. *F. avenaceum* co-existed together with *F. graminearum* and multiple regression analysis showed that both species negatively influenced pre-harvest quality factors of the crop such as specific weight. Furthermore, the non-toxigenic *Microdochium* species, which were found to strongly co-exist were also found to impact on the yield parameter TGW. To our knowledge, there are no previous reports on the effects of mixed populations of *Fusarium* and *Microdochium* spp. on yield parameters of malting barley.

### 4.2 Agronomy impact on FHB complex composition

Significant differences between regions and years for species composition were evident (Figs. 3 & 4), with higher concentrations of *Fusarium* spp. in the South and North of England and in Scotland in 2010 whilst no significant difference was observed in the Midlands between the two harvests. Analysis of the regional distribution of the two *Microdochium* species showed that the amount of *Microdochium* DNA was significantly higher in 2011 than in 2010 and significantly more *M. nivale* and *M. majus* were found in the North of England and in Scotland compared to the South or Midlands regions. The regional differences in the species composition of the FHB complex are possibly explained by the differences in their environmental requirements for growth and infection and climatic differences in the different regions. For example, in the North of England and Scotland average temperatures during the active growing season of the spring crop remain below 15 °C which is a more favourable environment for growth and infection by *Microdochium* species (Parry et al., 1995; Xu et al., 2008). In contrast, *F. poae* requires dry and warm conditions of around 25 °C for optimum growth (Parry et al., 1995; Xu et al., 2008). *F. graminearum* infection is more often associated with wet and warm conditions during anthesis, whereas *F. culmorum*, *F. avenaceum* and *F. tricinctum* require wet, humid and cool environmental conditions (Xu et al., 2008).

There were only small differences between the barley cultivars included in our studies with respect to the amounts of pathogen DNA present. The exception was cv Shuffle which had significantly lower amounts of total fungal DNA, irrespective of region, compared with the other elite cultivars such as Concerto, Forensic, Optic, Westminster (P = 0.042). This indicates that current commercially available cultivars, at least in the UK, are of similar susceptibility to *Fusarium* infection. Only a few sources of FHB resistance are known in barley, however, the level of resistance, even in these, is at best moderate (Bai and Shaner, 2004).

### 4.3 Mycotoxin contamination and safety of English and Scottish malting barley

Mycotoxin analysis of the UK barley samples revealed that the predominant mycotoxins were DON followed by NIV and ZON and lastly by HT-2 and T-2 at low concentrations. In 2010 and 2011 a large number of samples were analysed to obtain a representative overview of the natural mycotoxin contamination in English and Scottish fields and these were all found to be below the legislative limits of *Fusarium* related mycotoxins. In contrast to HT-2 and T-2, DON and NIV were found in significantly higher concentrations in 2011 than in 2010. The sum of HT-2 and T-2 found in the barley samples from 2010 was significantly associated with DNA of *F. langsethiae*. Besides *F. langsethiae*, *F. sporotrichioides* is also known to produce HT-2 and T-2 (Thrane et al., 2004). However in the UK, previous studies in oats have shown a strong relationship between combined HT-2 and T-2 levels and DNA amounts of *F. langsethiae* (Edwards et al., 2012), whereas in Europe three different species, *F. langsethiae*, *F. sporotrichoides* or *Fusarium sibiricum*, are associated with HT-2 and T-2 (Fredlund et al., 2010; Yli-Mattila et al., 2008, 2009). The barley samples were analysed for *F. sporotrichioides* DNA with primers known to cross-react with *F. sibiricum* (Yli-Mattila et al., 2011) but failed to detect the DNA of either species or to isolate any of these species from barley grain. Thus, the evidence suggests that in the UK, contamination with HT-2 and T-2 in both oats and barley is predominantly associated with *F. langsethiae*.

Isolates of *F. graminearum*, *F. culmorum* and *F. poae* are able to produce NIV; in the present study only *F. poae* correlated strongly (R^2^ = 0.84) with NIV concentrations. This finding is in agreement with previous studies in Northern Europe showing that *F. poae* is the main producer of NIV in barley (Bushnell et al., 2003; Nielsen et al., 2011; Yli-Mattila et al., 2004, 2008, 2009), and similar trends have been observed in UK oats (Edwards et al., 2012).

Samples collected from 2007 to 2009 were limited and not fully representative of these harvest years. These samples were selected on the range of their known mycotoxin concentrations and used to isolate, identify and quantify the main producers associated with mycotoxin accumulation. Of these samples only a single sample exceeded the legislative limit set for DON, a total of 19 samples of the 63 exceeded the legislative limit of ZON and none of the analysed samples exceeded the indicative limit of HT-2 and T-2. Regression analysis using individual DNA of *F. graminearum* or *F. culmorum* revealed that the relationship with DON and ZON was stronger for *F. graminearum*. However the regression for producers and mycotoxins was fitted best with cumulative data including *F. culmorum* DNA, suggesting that both species were implicated in the production of DON and ZON. The predominance of *F. graminearum* and *F. culmorum* as the main DON producers agrees with previous reports of strong correlations between the DNA of DON-producing *Fusarium* species and DON concentrations found in barley, wheat and oats (Fredlund et al., 2008; Nielsen et al., 2011; Sarlin et al., 2006; Waalwijk et al., 2004; Yli-Mattila et al., 2008, 2009, 2011). In two samples, low levels of DON were detected but the DNA of *F. graminearum* or *F. culmorum* were below the level of quantification, thus it is possible that DON in these two samples may have been associated with different producers, for example *Fusarium equiseti* and/or *Fusarium acuminatum* which are also known DON producers in cereals in Europe (Marín et al., 2012).

### 4.4 Impact of FHB pathogens on malting and brewing quality parameters

The major species to significantly affect quality parameters in further micromalting studies were identified as *M. nivale*, *F. poae* and *F. langsethiae*. Although the survey samples from 2010 to 2011 were randomly selected from sites across the UK (and thus representative), it should be noted that barley with GE (4 ml) counts of less than 98% would not be processed in commercial malting. In the present experiment, the GE (4 ml) requirement was relaxed to > 80%, in order to include samples in the survey which had more widely ranging contents of the *Fusarium* and *Microdochium* species investigated. These samples would not normally have been malted for brewing use. However, in the majority of cases, the resultant malt friability and α-amylase values confirmed that samples had malted satisfactorily. Thus, the GE of these samples had declined through storage, but the germinative capacity (percentage of live grains) was still sufficient.

The micromalting experiment was not balanced in terms of the numbers of each cultivar malted in each year. This was largely due to the availability of samples within the survey which had sufficient germinative energy to malt and which showed interesting variations with regard to their measured concentrations of fungal DNA and mycotoxins. In general the malts prepared were of acceptable specification (although precise requirements depend on the end user). If anything, the majority of malts were rather well modified (friability > 90% and with high α-amylase activities), which was a result of the generous 50 h steep cycle, designed to ensure that barley samples of differing provenance would all hydrate and modify sufficiently.

Water sensitivity is defined as the difference between the GE (4 ml) and GE (8 ml) counts. The number (expressed as a percentage) indicates whether a malt sample has lower germinative energy in the presence of excess water. In the present study, both *M. nivale* and *F. poae* were significant factors which correlated positively with water sensitivity. Crop year was also a significant factor in determining water sensitivity, with 2011 samples having on average, greater water sensitivity than those from 2010. Water sensitivity is of commercial significance because the maltster will need to adjust the steeping process (e.g. the duration of air rests) when malting water sensitive grain. Water sensitivity has been linked to malt microflora (Woonton et al., 2005) although other factors seem to be involved, as treatment of grains with anti-microbial agents does not consistently overcome water sensitivity (Kelly and Briggs, 1992). The fact that water sensitivity was also affected by crop year could be caused by differences in climatic/agronomic influences during the respective years. It could also reflect the fact that on average more fungal DNA was found in the 2011 samples for the two species identified as being significant in the model for water sensitivity (0.027 pg/ng as compared with 0.015 pg/ng for *F. poae* and 0.37 pg/ng versus 0.19 pg/ng for *M. nivale*).

There was a positive correlation of *F. poae* with wort FAN suggesting that *F. poae* contributes to proteolytic activity through the malting and mashing processes, thus increasing FAN production, particularly during the low temperature stand at 45 °C during the congress mash schedule. The model for wort FAN also included *F. langsethiae* and an interaction term between the two species. The interaction indicated that at low concentrations of *F. langsethiae*, *F. poae* dominated with regard to increasing wort FAN, whereas at high *F. langsethiae* concentrations and low *F. poae*, the contribution to FAN from *F. langsethiae* was significant. The trends found in the interactions of *F. poae*, *F. langsethiae* and wort FAN may reflect competitive aspects between the growth habits of these two species. These results are consistent with prior reports of protease secretion by *F. poae* (Pekkarinen et al., 2000; Schwarz et al., 2002). Pekkarinen et al. (2000) reported that *F. poae* grown on barley grains produced non-specific protease activities at pH 5.0 (close to mash pH) and pH 8.0, over a period of days following inoculation. These activities were not present in non-inoculated barley. Schwarz et al. (2002) conducted a glasshouse trial where barley plots were inoculated separately with *F. graminearum* and *F. poae*. The high wort FAN contents reported for the inoculated plots led the authors to conclude that *Fusarium* spp. contributed exoproteinase as well as endoproteinase activities.

The results presented here suggest that *M. nivale* can have a significant impact upon the quality of malting barley. On balance, these impacts were undesirable as, although positively correlated with friability, *M. nivale* also correlated with increased water sensitivity, lower germinative energy and had a negative impact on the laboratory wort filtration volume. The latter is a crude predictor of the mash separation performance of malt in a brewhouse (Evans et al., 2011). A lower volume of filtered wort after the specified time interval indicates that the mash might take longer to filter on a commercial scale. Although the model for wort filtration volume was significant, it had a low predictive power, indicating that many other variables not accounted for in the present study can influence mash separation performance. *M. nivale* occurrence, or prevalence in the FHB complex, has been associated with regions experiencing relatively cool temperatures and frequent, short, showers (Doohan et al., 2003; Nielsen et al., 2011).

The absence of any direct relationship between the presence of *Fusarium* spp. and *Microdochium* spp. and wort viscosity was contrary to previous reports of a reduction in wort viscosity in *Fusarium*-infected malts, which was attributed to glucanase and xylanase activities of *Fusarium* spp. (Schwarz et al., 2002). However, a recent study reported increases in wort β-glucans when brewing with malts prepared from grain artificially inoculated with *F. culmorum* (Oliveira et al., 2012a). Hence the precise impact of infection may depend on the particular β-glucanase activities present and the mashing schedule employed. β-glucan solubilase activity will solubilise high molecular weight β-glucans during mashing, thus tending to increase wort viscosity. Endo-β-glucanase activities then reduce the mean molecular weight of glucans present and thus act to reduce wort viscosity. It is further true that in most prior studies control malts were compared with artificially inoculated barley malts, whereas in the present trial we investigated natural variations in the grain microflora from survey sites across the UK.

Wort colour is determined to a large extent during kilning. Since the same kiln temperature cycle was used for all samples, colour differences were caused principally by variations in concentrations of the Maillard browning reaction precursors (reducing sugars and free amino nitrogen) present following germination. It is consistent in this regard that increasing *M. nivale* DNA correlated both with increasing malt friability and laboratory wort colour, since the release of amino acids and reducing sugars from the breakdown of protein and starch respectively, increases with the extent of modification and friability of malt.

There have been several reports on the changes in the diversity of composition of the FHB complex in cereals within different geographical and climatic locations. In the past ten years in Europe, *F. culmorum* has been replaced by *F. graminearum* (Waalwijk et al., 2003; Jennings et al., 2004; Stepien et al., 2008; Xu et al., 2008; Isebaert et al., 2009; Nielsen et al., 2011) and furthermore *F. poae* has been shown to replace *F. graminearum* in Southern Europe (Pancaldi et al., 2010; Shah et al., 2005). In contrast, in Central Europe and in North America and China, DON is the main trichothecene associated predominantly with *F. graminearum* and species of the *F. graminearum* clade (O'Donnell et al., 2004).

In these studies we describe the impact of newly emerging species of importance, *M. nivale* and *F. langsethiae*, on the malting and brewing quality of naturally infected barley. The results clearly indicate that the pathogen populations of the FHB complex in barley in the UK are diverse and dominated by non-toxigenic *Microdochium* species and toxigenic *Fusarium* species such as *F. poae*, *F. avenaceum* and *F. langsethiae*. Future research efforts should focus on elucidating the impact of these newly emerging species and their mycotoxins, for example, enniatins produced by *F. avenaceum* and *F. tricinctum* on barley and HT-2 and T-2 produced by *F. langsethiae*.

## Figures and Tables

**Table A.1:** Total number of survey samples included in the survey from 2007 to 2011 distribution of samples used in mycotoxin and brewing quality analysis.

Year	Total samples	Analysis	
		Mycotoxin	Brewing
2007	12	12	
2008	30	30	
2009	21	21	
2010	89	35	27
2011	76	45	27
Total	228	143	54

**Table A.2:** Survey samples from 2010 to 2011 divided on regions and varieties.

Year	Total samples	Region				Varieties								
		South	Midlands	North	Scotland	Concerto	Forensic	Optic	Propino	Quench	Shuffle	Tipple	Westminster	Other
2010	89	5	20	28	29	6	5	17	4	20	0	20	6	4
2011	76	25	27	6	15	16	0	11	6	19	6	13	0	2
Total	228	30	47	34	44	22	5	28	10	39	6	33	6	6

**Table A.3:** Sequences, efficiency and references of primers used for quantitative real-time PCR.

Target	Primer name	Sequence (5′–3′)	Efficiency (%)	Reference
*F. graminearum*	Fg16NF Fg16NR	ACAGATGACAAGATTCAGGCACA TTCTTTGACATCTGTTCAACCCA	97%	Nicholson et al. (1998), Brandfass and Karlovsky (2008)
*F. culmorum*	Fc01F Fc01R	ATGGTGAACTCGTCGTGGC CCCTTCTTACGCCAATCTCG	100%	Nicholson et al. (1998)
*F. langsethiae*	FlangF3 LanspoR1	CAAAGTTCAGGGCGAAAACT TACAAGAAGACGTGGCGATAT	97%	Wilson et al. (2004), Edwards et al. (2012)
*F. avenaceum*	Fave574 fwd Fave627 rev	TATGTTGTCACTGTCTCACACCACC AGAGGGATGTTAGCATGATGAAG	103%	Nicolaisen et al. (2009)
*F. tricinctum*	Ftri573 fwd Ftri630 rev	TTGGTATGTTGTCACTGTCTCACACT T TGACAGAGATGTTAGCATGATGCA	99%	Nicolaisen et al. (2009)
*F. poae*	FpoaeA51 fwd FpoaeA98 rev	ACCGAATCTCAACTCCGCTTT GTCTGTCAAGCATGTTAGCACAAGT	99%	Nicolaisen et al. (2009)
*F. sporotrichioides*	Fspor F1 LansporR1	CGCACAACGCAAACTCATC TACAAGAAGACGTGGCGATAT	95%	Wilson et al. (2004), Edwards et al. (2012)
*M. nivale*	Mniv1f Mniv1r	TTGGCTTGCACAAACAATACTTTTT AGCACAACAGGCGTGGATAAG	99%	Nielsen et al. (2013)
*M. majus*	Mmajus1f Mmajus1r	AACCCCTCCCGGGTCAG GGATAAACGACACTTGAAGACAGAAAA	99%	Nielsen et al. (2013)

**Table 1:** DNA of *Fusarium* spp. and *Microdochium* spp. (pg/ng of total DNA) of UK malting barley samples collected in 2007 to 2011 described by mean value, 95% confidence interval and incidence (%).

Year	n	Fungal DNA (pg/ng of total DNA) mean ± 95% confidence interval (incidence %)							
		Fl	Fp	Fg	Fc	Fa	Ft	Mn	Mm
2007	12	0.003 ± 0.002	0.341 ± 0.119	0.087 ± 0.059	0.075 ± 0.035	0.112 ± 0.037	0.098 ± 0.125	0.567 ± 0.211	3.843 ± 1.540
		(58)	(100)	(100)	(92)	(100)	(75)	(100)	(100)
2008	30	0.012 ± 0.07	0.602 ± 0.287	0.389 ± 0.442	0.040 ± 0.022	0.249 ± 0.142	0.042 ± 0.040	0.870 ± 0.421	1.074 ± 0.328
		(62)	(100)	(83)	(48)	(100)	(41)	(100)	(100)
2009	21	0.030 ± 0.02	1.019 ± 0.997	0.129 ± 0.076	0.191 ± 0.208	0.462 ± 0.598	0.128 ± 0.086	1.081 ± 0.945	0.863 ± 0.455
		(52)	(100)	(76)	(62)	(100)	(67)	(90)	(100)
2010	75	0.017 ± 0.05	0.012 ± 0.005	0.0002 ± 0.0002	0.024 ± 0.012	0.040 ± 0.022	0.098 ± 0.047	0.137 ± 0.039	0.448 ± 0.239
		(80)	(80)	(15)	(28)	(100)	(85)	(93)	(81)
2011	76	0.005 ± 0.002	0.020 ± 0.009	0.005 ± 0.002	0.002 ± 0.001	0.023 ± 0.007	0.021 ± 0.008	0.415 ± 0.120	0.542 ± 0.261
		(54)	(92)	(50)	(24)	(99)	(96)	(97)	(91)

LOQ: 0.0001 pg/ng of total DNA. Abbreviations: number of samples (n), *F. langsethiae* (Fl), *F. poae* (Fp), *F. graminearum* (Fg), *F. culmorum* (Fc), *F. avenaceum* (Fa), *F. tricinctum* (Ft), *M. nivale* (Mn), and *M. majus* (Mm).

**Table 2:** T-2 and HT-2, nivalenol (NIV), deoxynivalenol (DON) and zearalenone (ZON) in UK malting barley samples collected in 2007 to 2011 described by mean value, 95th percentile and maximum detected value.

Year	n	Mycotoxin concentration (μg/kg)														
		T-2			HT-2			NIV			DON			ZON		
		Mean	95th%	Max	Mean	95th%	Max	Mean	95th%	Max	Mean	95th%	Max	Mean	95th%	Max
2007	12	5	5	5	5	5	5	24	42	47	283	816	974	75	202	214
2008	30	6	5	24	5	5	19	22	98	206	211	672	3599	192	1148	1558
2009	21	12	24	130	7	19	27	34	112	122	134	594	707	95	360	1116
2010	35	9	24	52	17	57	87	26	68	209	14	56	66	2	4	4
2011	45	5	5	22	5	8	11	180	853	1089	56	195	255	3	5	50

LOQ: T-2 = 10 μg/kg, HT-2 = 10 μg/kg, NIV = 10 μg/kg, DON = 10 μg/kg, and ZON = 2 μg/kg. The data was corrected for recovery and not detected samples were assigned half the LOQ value. Number of samples (n).

**Table 3:** Multiple linear regression with groups for year of FHB related species on thousand grain weight (TGW) (g) and specific weight (SW) (kg/hl) in UK malting barley samples collected from 2007 to 2011 with sufficient grain numbers available for analysis. Regression of TGW on DNA of *M. majus*, *M. nivale* and *F. avenaceum* was significant and fitted separate, non-parallel lines for each individual year. Regression of SW on DNA of *F. avenaceum* and *F. graminearum* fitted separate but parallel lines for each year.

Year	Equation	
	TGW (g)	SW (kg/hl)
2007	y = 32.25 + 2.10 logFa − 12.8 logMn + 11.0 log Mm	y = 57.79 − 1.172 logFa − 0.892 logFg
2008	y = 42.24 − 2.44 logFa − 3.97 logMn + 6.05 log Mm	y = 62.17 − 1.172 logFa − 0.892 logFg
2009	y = 47.13 + 0.46 logFa − 1.94 logMn − 1.68 log Mm	y = 61.83 − 1.172 logFa − 0.892 logFg
2010	y = 47.49 − 1.98 logFa + 2.76 logMn − 0.77 log Mm	y = 63.60 − 1.172 logFa − 0.892 logFg
2011	y = 38.39 − 2.54 logFa + 2.70 logMn − 3.38 log Mm	y = 62.77 − 1.172 logFa − 0.892 logFg
	d.f. = 177, R^2^ = 0.40, P < 0.001	d.f. = 64, R^2^ = 0.48, P < 0.001

**Table 4:** Summary data for malt and wort quality parameters, based on a total of 54 micromalted barley samples and broken down according to barley variety and season described by mean and 95% confidence interval. Abbreviations: number of samples (n) and germinative energy (GE).

Variety	Year	n	GE (4 ml)	Malt friability (%)	Malt α-amylase (DU)	Malt β-amylase (betamyl units)	Hot water extract (congress mash; %)	Lab wort filtration volume (ml)	Free amino nitrogen (mg/l)	Lab wort viscosity (mPa·s)	Lab wort colour (EBC)
Optic	2010	6	94.7 ± 1.4	95.1 ± 2.2	73.3 ± 10.1	17.4 ± 2.0	82.6 ± 0.29	259 ± 19.7	179 ± 22.4	1.45 ± 0.05	3.74 ± 0.28
	2011	5	95.2 ± 2.3	93.3 ± 3.6	69.1 ± 8.1	17.8 ± 2.6	82.3 ± 0.73	277 ± 27.8	179 ± 11.6	1.46 ± 0.07	3.76 ± 0.50
Quench	2010	6	94.8 ± 1.9	95.1 ± 3.0	73.2 ± 7.1	16.0 ± 1.2	82.4 ± 0.76	234 ± 23.2	191 ± 11.5	1.45 ± 0.03	3.89 ± 0.24
	2011	11	93.7 ± 2.5	89.7 ± 4.0	60.9 ± 5.8	14.4 ± 2.5	81.4 ± 0.86	241 ± 28.6	189 ± 17.4	1.49 ± 0.04	4.14 ± 0.36
Tipple	2010	15	92.3 ± 3.0	83.6 ± 4.0	82.1 ± 6.4	18.2 ± 1.5	81.0 ± 0.91	242 ± 17.8	190 ± 9.9	1.42 ± 0.02	3.55 ± 0.18
	2011	11	91.9 ± 2.5	68.6 ± 12.3	65.4 ± 6.0	18.6 ± 1.8	81.1 ± 0.84	245 ± 16.7	185 ± 13.2	1.51 ± 0.04	3.67 ± 0.30

Abbreviations: number of samples (n) and germinative energy (GE).

**Table 5:** Significant factors and their impacts on barley/malt quality parameters.

Barley/malt quality parameter	Significant factors (p-value) and their impact on the specified parameter (+ or −)					Model R^2^	Model prob. > F
	*F. poae*	*F. langsethiae*	*M. nivale*	Variety	Year		
Germinative Energy (4 mL)	0.0198 (−)	–	0.0007 (−)	–	–	0.24	0.0010
Water Sensitivity	0.0388 (+)	–	0.0007 (+)	–	< 0.0001 (2011 > 2010)	0.58	< 0.0001
Malt Friability	–	–	0.0023 (+)	< 0.0001 (Q = O > T)	0.0030 (2010 > 2011)	0.48	< 0.0001
Malt alpha amylase (DU)	–	–	–	–	0.0006 (2010 > 2011)	0.21	0.0006
Extract of malt (congress mash/fine grind, %)	0.0103 (−)	0.0048 (−)	–	–	–	0.28	0.0003
Lab wort filtration vol/25 min	–	–	0.0073 (−)	0.0145 (T = Q < O)	–	0.26	0.0022
Free amino nitrogen (mg/l)	0.0003* (+)	0.0402* (+)	–	–	–	0.39	< 0.0001
Lab wort colour	–	–	< 0.0001 (+)	0.0067 (Q > O = T)	–	0.58	< 0.0001

Abbreviations: Tipple (T), quench (Q), and optic (O), *Significant interaction term between *F. poae* and *F. langsethiae*, P = 0.0073. At low *F. langsethiae*, *F. poae* increased free amino nitrogen.
